# Gender heterogeneity in dyslipidemia prevalence, trends with age and associated factors in middle age rural Chinese

**DOI:** 10.1186/s12944-020-01313-8

**Published:** 2020-06-12

**Authors:** Minmin Wang, Mengfei Liu, Fenglei Li, Chuanhai Guo, Zhen Liu, Yaqi Pan, Ying Liu, Fangfang Liu, Hong Cai, Yangfeng Wu, Zhonghu He, Yang Ke

**Affiliations:** 1 Key Laboratory of Carcinogenesis and Translational Research (Ministry of Education/Beijing), Laboratory of Genetics, Peking University Cancer Hospital and Institute, Beijing, China; 2 Hua County People’s Hospital, Anyang, China; 3 Peking University Clinical Research Institute, Beijing, China

**Keywords:** Lipid management, Gender heterogeneity, Dyslipidemia, Epidemiology, Lipids and lipoproteins, Prevalence, Risk factor

## Abstract

**Background:**

Heterogeneity should be carefully addressed to facilitate establishment of effective population-level blood lipid management. The primary aim of the study was to investigate gender heterogeneity in prevalence of dyslipidemia, including trends with age and associated factors in middle age rural Chinese.

**Methods:**

This is a cross-sectional study based on a baseline investigation of a population-based randomized controlled trial in rural China, involving 26,378 permanent residents of age 45–69. The age-specific prevalence of dyslipidemia was estimated for men and women, and the trends of prevalence with age were compared. Logistic regression was used to explore the factors associated with prevalent risk of dyslipidemia.

**Results:**

The overall prevalence of dyslipidemia was significantly higher in females than in males for borderline high and above (BHA) total cholesterol (TC ≥ 200 mg/dL), BHA triglycerides (TG ≥ 150 mg/dL) and BHA low-density lipoprotein cholesterol (LDL-C ≥ 130 mg/dL), but was lower for low high-density lipoprotein cholesterol (HDL-C < 40 mg/dL) in females than the corresponding prevalence in males. The prevalence of borderline high and above TC, TG and LDL-C all rose with age in females, but was stable or even decreased with age in males. In contrast, graphic representation of the prevalence of low HDL-C showed no striking age related trend in both genders. Risk of dyslipidemia was associated predominantly with obesity in males, but was more predominantly associated with hypertension in females.

**Conclusion:**

Heterogeneity was found in comparing the prevalence of dyslipidemia in men and women, and gender heterogeneity was found in its trend with age and associated factors in middle aged rural Chinese. The effectiveness of population-level blood lipid management and CVD primary prevention programs in China is expected to be improved if gender heterogeneity is considered.

## Introduction

Blood lipids are fatty substances free in the blood or bound to other molecules. Lipids play a significant role in energy storage, cell membrane structure, cytokine synthesis, signal transmission and overall basal metabolism [1]. Within numerous different lipids and lipoproteins, triglycerides (TG), total cholesterol (TC), and the two major components of TC namely low-density lipoprotein cholesterol (LDL-C) and high-density lipoprotein cholesterol (HDL-C), are the most commonly used indicators and control targets in clinical settings and population-level intervention projects.

Dyslipidemia is defined by an abnormal profile of blood lipids [2, 3], and dyslipidemia contributes significantly to atherosclerosis [4]. Elevated levels of plasma LDL-C, TC and TG have been shown to be crucial risk factors for cardiovascular disease (CVD) [5–7]. Basic data regarding prevalence and factors associated with these lipid indicators provides a foundation for establishing strategies for blood lipid management, and this is a key step in the primary prevention of CVD [8].

To date, several nation-wide population-based studies have been conducted in urban and rural areas to investigate the prevalence of dyslipidemia in Chinese adults. Previous studies have reported differing gender-specific prevalence in dyslipidemia in China [9–16]. For example, the 2010 Chinese Chronic Disease Survey which was based on data from 162 surveillance points (59,001 rural residents over 18 years) in 31 provinces, reported the prevalence of high TC, high TG, high LDL-C and low HDL-C were 3.0, 13.0, 1.8, 49.5% for males, and 2.7, 8.7, 1.7, 39.5% for females in rural China [14]. However, the potential heterogeneity in prevalence of dyslipidemia in males vs. females has not been systematically evaluated.

A pilot blood lipid management project has been underway in China since 2013, and a nation-wide blood lipid management project is going to be initiated in the near future [17]. In this project, blood lipid management is targeted to the whole population, under the assumption that heterogeneity does not exist among subgroups of subjects [18, 19]. One potential pitfall of using this “universal” strategy is the imprecision and lack of prioritizing in defining populations to be intervened. This defect would be greatly enlarged in case that heterogeneity of dyslipidemia prevalence exists in subgroup(s) of subjects with particular demographic characteristics. The heterogeneity of prevalence and risk factor profiles of dyslipidemia should be carefully addressed to facilitate establishment of effective population-level blood lipid management and a CVD primary prevention strategy.

In this study, the prevalence of dyslipidemia in TC, TG, LDL-C and HDL-C was investigated in a rural Chinese population based on a large-scale population-based randomized controlled trial. The prevalence and trends with age for these four commonly used indicators in men vs. women were evaluated, and the heterogeneity in risk factor patterns for dyslipidemia in males and females was assessed.

## Methods

### Parent study

In 2012, the Endoscopic Screening for Esophageal Cancer in China (ESECC) randomized controlled trial (ClinicalTrials.gov identifier: NCT01688908) [20] was initiated in Hua County, which aimed to evaluate the efficacy and cost-effectiveness of endoscopic screening for Esophageal Squamous Cell Carcinoma. The design and preliminary results of this ESECC trial can be found elsewhere [20]. Briefly, 668 out of 846 villages in rural Hua County with population size ranging from 500 to 3000 were randomly selected and allocated into the intervention or control arm of the study at a ratio of 1:1, using a blocked randomization procedure based on the total population size of each village. In the baseline investigation of this ESECC trial, a total of 35,772 permanent residents aged 45–69 in these target villages signed an informed consent form to participate and provided blood samples. This included about 20% of all eligible residents in the target villages.

### Study design and participants

The current study is a cross-sectional survey based on baseline investigation data of the ESECC trial. Eligibility criteria for this study included: 1) permanent residency in a target village; 2) age 45–69; 3) signed informed consent for participation; 4) questionnaire completed; 5) blood samples provided, with valid test results for blood lipids. A total of 26,378 subjects in the baseline investigation of the ESECC trial were enrolled in this study (see Supplementary Figure 1).

### Physical examination and questionnaire interview

All participants in this study received a measurement of height and weight in baseline investigation. Participants’ seated blood pressure was measured once after 5 min of rest with mercury sphygmomanometer. A computer-aided one-on-one questionnaire was then conducted by well-trained interviewers to collect information on personal characteristics (e.g. age and gender) and socioeconomic status (level of education, family size, work type, annual income per capital and marital status), lifestyle factors (cigarette smoking, alcohol consumption, source of drinking water and intake of fruit, vegetable, protein, fried food, salty food and spicy food) and health status (self-reported CVD history, history of diabetes and symptoms of heartburn and regurgitation). Detailed definitions and coding for potential risk factors of dyslipidemia evaluated in this study are listed in Supplementary Table 1.

### Blood lipid measurement

At physical examination, a fasting blood sample of ~ 5 mL was collected from each participant in a heparin sodium anticoagulant tube. These tubes were then centrifuged at 1000 rpm for 5 min and the supernatant was sent for lipid measurement of TC, LDL-C, HDL-C and TG within 4 h in the clinical laboratory of Hua County People’s Hospital. Lipid measurements were conducted using a HITACHI7600 automatic biochemistry analyzer (Hitachi High Technologies Co., Tokyo, Japan) with commercially available reagents (Autobio Diagnostics Co., Ltd., Beijing, China) for analysis of TC, TG, LDL-C and HDL-C.

### Definition of dyslipidemia

**Fig. 1:**
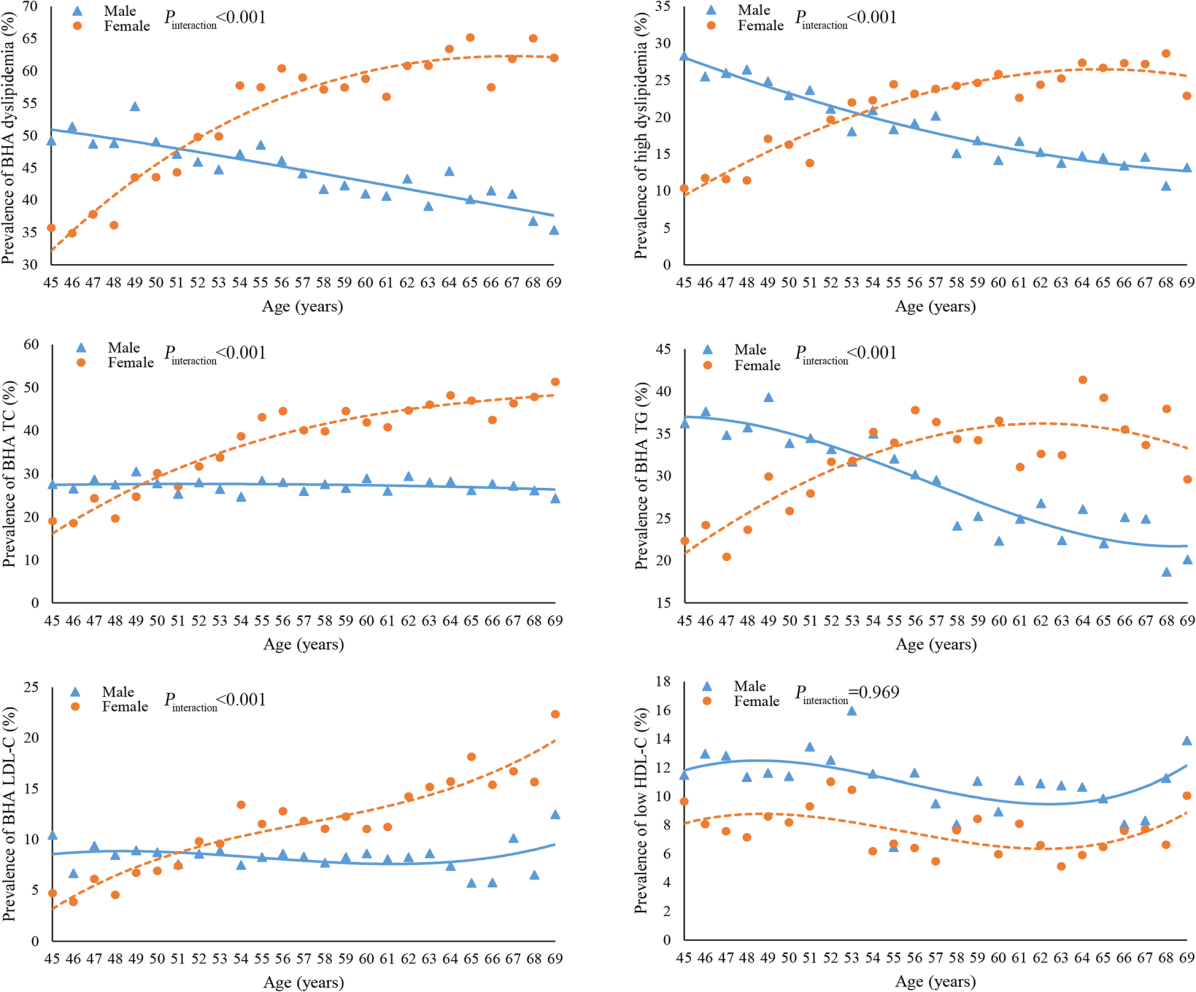
The age and gender distribution of dyslipidemia prevalence in 26,378 participants from rural Hua County, China, 2012–2016 ^a^. **a** Age and gender distribution of borderline high and above (BHA) dyslipidemia. **b** Age and gender distribution of high dyslipidemia. **c** Age and gender distribution of BHA total cholesterol (TC). **d** Age and gender distribution of BHA triglycerides (TG). **e** Age and gender distribution of BHA low-density lipoprotein cholesterol (LDL-C). **f** Age and gender distribution of low levels of high-density lipoprotein cholesterol (HDL-C). ^a^ A cubic polynomial curve was fitted to reduce random fluctuations. Heterogeneity between genders was tested using models with main effects and interaction terms of gender variable and the linear, quadratic and cubic forms of age variable separately. The *P* values presented were under the null hypothesis that “the coefficients of all interaction terms equal zero”

According to the guidelines for the prevention and treatment of dyslipidemia in Chinese adults (2016 version) [3], borderline high TC was defined as ≥200 mg/dL (5.2 mmol/L) and < 240 mg/dL (6.2 mmol/L); high TC as ≥240 mg/dL (6.2 mmol/L); borderline high TG as ≥150 mg/dL (1.7 mmol/L) and < 200 mg/dL (2.3 mmol/L); high TG as ≥200 mg/dL (2.3 mmol/L); borderline high LDL-C as ≥130 mg/dL (3.4 mmol/L) and < 160 mg/dL (4.1 mmol/L); high LDL-C as ≥160 mg/dL (4.1 mmol/L); low HDL-C as < 40 mg/dL (1.0 mmol/L). In consideration of discrepancies in biologic function and the notable heterogeneity of age distribution between HDL-C and the other three indicators (see Fig. 1), dyslipidemia was defined on the basis of only TC, TG and LDL-C. Two sets of definitions of dyslipidemia were used in this study, namely borderline high and above (BHA) dyslipidemia, and high dyslipidemia. BHA dyslipidemia was defined as the presence of a high level or borderline high level of any factor including TC, TG or LDL-C, and high dyslipidemia was defined as the presence of a high level of any one of these three lipid factors in a given study subject.

### Statistical analysis

The Chi-square test and Student’s t test were used to compare demographic characteristics and behavioral factors in ESECC participants who were enrolled and not enrolled, as well as among male and female subjects included in this study. Gender stratified prevalence of lipid abnormalities was estimated for TC, TG, LDL-C, HDL-C, BHA dyslipidemia and high dyslipidemia, and difference in the distribution of these prevalence estimates in males vs. females was assessed using the Chi-square test for dichotomous variables and Ridit analysis for multi-categorical variables. For age related trends, a cubic polynomial curve was applied to reduce random fluctuations, and the significance of heterogeneity was tested by fitting models with age and gender and the interaction terms of age and gender variables. Univariate and multiple unconditional logistic regression models were applied to identify factors associated with ‘borderline high and above (BHA) dyslipidemia’ and ‘high dyslipidemia’ separately. First a multiple model was constructed based on all the participants, and gender heterogeneity in the effect of each risk factor was tested by adding the interaction term of the specific risk factor (one term at a time) and gender variable into the final model. As significant interactions between important behavior factors and gender were found (see below), risk factors were identified separately in males and females with logistic regression analysis. Statistical analysis was performed using STATA (Version 13.1; Stata Corp LLC, TX, USA). All tests were two-sided and *P* values < 0.05 were considered to be statistically significant.

## Results

Key demographic characteristics and behavior variables were compared between ESECC participants enrolled in and excluded from this study (Supplementary Table 2). The distribution of age, gender, job, smoking, alcohol consumption, fried food intake, spicy food intake and self-reported history of diabetes were similar between the two groups. Whereas there were differences between the two groups for education, BMI, blood pressure, water source and heartburn and regurgitation. Of the 26,378 subjects enrolled in this study, 12,925 (49.00%) were men and 13,453 (51.00%) were women. Compared with females, male participants were slightly older, more likely to have a non-physical job, more likely to smoke, consumed more alcohol, were more likely to have hypertension, ate more fried and spicy food, and a higher proportion of these individuals had heartburn and regurgitation. The proportion of individuals with obesity and a self-reported history of diabetes was higher in females than that in males. In evaluation of the four lipid indicators, females had significantly higher levels of TC, LDL-C and HDL-C compared with male participants (*P* < 0.001). TG did not show a statistically significant difference in genders (Table 1).

**Table 1:** Selected demographic and behavioral characteristics in 26,378 individuals from rural Hua County, China, 2012–2016

	Participants in this study (*N* = 26,378) n (%)	Male (*N* = 12,925) n (%)	Female (*N* = 13,453) n (%)	*P* value ^a^
Age				
Mean (SD)	56.52 (6.88)	56.84 (6.87)	56.21 (6.88)	< 0.001
Educational level				
Illiterate	8537 (32.36)	1901 (14.71)	6636 (49.33)	< 0.001
Primary or Middle School	15,252 (57.82)	9139 (70.71)	6113 (45.44)	
High school or above	2589 (9.81)	1885 (14.58)	704 (5.23)	
Job				
Physical labor	25,909 (98.22)	12,544 (97.05)	13,365 (99.35)	< 0.001
Nonphysical work	469 (1.78)	381 (2.95)	88 (0.65)	
Body mass index ^c^				
≤ 24.0 kg/m^2^	9808 (37.18)	4811 (37.22)	4997 (37.14)	0.013
24.1–27.9 kg/m^2^	10,869 (41.20)	5394 (41.73)	5475 (40.70)	
≥ 28.0 kg/m^2^	5618 (21.30)	2670 (20.66)	2948 (21.91)	
Unknown ^b^	83 (0.31)	50 (0.39)	33 (0.25)	
Blood pressure ^d^				
No hypertension	12,019 (45.56)	5582 (43.19)	6437 (47.85)	< 0.001
Hypertension	14,212 (53.88)	7260 (56.17)	6952 (51.68)	
Unknown ^b^	147 (0.56)	83 (0.64)	64 (0.48)	
Water source ^e^				
Deep well	17,475 (66.25)	8553 (66.17)	8922 (66.32)	0.802
Shallow well or other	8903 (33.75)	4372 (33.83)	4531 (33.68)	
Smoking ^f^				
None	17,269 (65.47)	3968 (30.70)	13,301 (98.87)	< 0.001
Moderate	6797 (25.77)	6657 (51.50)	140 (1.04)	
Large amount	2265 (8.59)	2255 (17.45)	10 (0.07)	
Unknown ^b^	47 (0.18)	45 (0.35)	2 (0.01)	
Alcohol consumption ^g^				
None	20,569 (77.98)	7196 (55.68)	13,373 (99.41)	< 0.001
Moderate	4351 (16.49)	4277 (33.09)	74 (0.55)	
Large amount	1445 (5.48)	1441 (11.15)	4 (0.03)	
Unknown ^b^	13 (0.05)	11 (0.09)	2 (0.01)	
Fried food intake				
Seldom	18,767 (71.15)	8843 (68.42)	9924 (73.77)	< 0.001
Often	7611 (28.85)	4082 (31.58)	3529 (26.23)	
Spicy food intake				
Seldom	16,888 (64.02)	8093 (62.62)	8795 (65.38)	< 0.001
Often	9490 (35.98)	4832 (37.38)	4658 (34.62)	
Heartburn and regurgitation				
No	19,193 (72.76)	9220 (71.33)	9973 (74.13)	< 0.001
Yes	7185 (27.24)	3705 (28.67)	3480 (25.87)	
Self-reported history of diabetes				
No	25,834 (97.94)	12,684 (98.14)	13,150 (97.75)	0.027
Yes	544 (2.06)	241 (1.86)	303 (2.25)	
TC level (mg/dL) ^h^				
Mean (SD)	186.49 (35.67)	182.26 (34.90)	190.55 (35.93)	< 0.001
TG level (mg/dL) ^i^				
Mean (SD)	139.46 (111.86)	140.20 (129.57)	138.76 (91.68)	0.297
LDL-C level (mg/dL) ^h^				
Mean (SD)	97.67 (25.27)	95.95 (24.84)	99.32 (25.57)	< 0.001
HDL-C level (mg/dL) ^h^				
Mean (SD)	52.73 (14.08)	51.92 (14.26)	53.50 (13.86)	< 0.001

^a^ The Chi-square test and Student’s t test were used to compare demographic characteristics and behavioral factors in male and female subjects included in this study

^b^ The “Unknown” category was not included in the analysis

^c^ BMI was calculated as body weight in kilograms divided by the square of body height in meters (kg/m^2^). Subjects were categorized into three groups as BMI ≤ 24.0 (normal, coded as 0), 24.0 < BMI < 28.0 (overweight, coded as 1) and BMI ≥28.0 (obesity, coded as 2)

^d^ Blood pressure was measured for each participant. Subjects with systolic blood pressure ≥ 140 mmHg or diastolic blood pressure ≥ 90 mmHg were defined as hypertensive and coded as 1; others were defined as non-hypertensive and coded as 0

^e^ Participants were asked about their drinking water source in the questionnaire. This question had two options: 1) deep well (> 100 m); 2) shallow well or others (≤100 m). Subjects selecting 1) were coded as 0, and subjects selecting 2) were coded as 1

^f^ Participants were asked whether they smoked, if so over what period of time, and quantity of smoking in the questionnaire. Total amounts of cigarette consumption were calculated as time period multiplied by quantity of smoking. The smoking group was categorized by quartiles of accumulative consumption. Subjects who didn’t smoke were coded as 0; subjects with consumption in Q1-Q3 were defined as moderate-smokers and coded as 1; subjects with consumption in Q4 were defined as heavy-smokers and coded as 2

^g^ Participants were asked whether they had drunk alcohol and if so over what period of time and in what quantity in the questionnaire. Total amounts of alcohol consumption were calculated as period of time multiplied by quantity of drinking. The alcohol drinking groups were categorized by quartiles of accumulative consumption. Subjects who didn’t drink were coded as 0; subjects with consumption in Q1-Q3 were defined as moderate drinkers and coded as 1; subjects with consumption in Q4 were defined as heavy drinkers and coded as 2

^h^ To convert cholesterol to mmol/L, multiply values by 0.0259

^i^ To convert triglycerides to mmol/L, multiply values by 0.0113

Table 2 shows prevalence of lipid abnormalities stratified by gender (detailed gender- and age-specific prevalence are recorded in Supplementary Table 3). The prevalence of borderline high TC, high TC, borderline high TG, high TG, borderline high LDL-C, high LDL-C and low HDL-C in this rural population was 24.53, 7.50, 15.89, 14.53, 7.81, 1.76, and 9.19% respectively. For dyslipidemia as defined in this study, the prevalence of BHA dyslipidemia and high dyslipidemia was 48.68 and 19.90% respectively. Regarding the heterogeneity between genders, prevalence estimates of all the lipid indicators showed significant difference in males and females. Specifically, females had a higher proportion of abnormalities in TG, TC and LDL-C, but a lower proportion of HDL-C abnormalities compared with the corresponding prevalence in males (Table 2).

**Table 2:** Gender-specific prevalence of dyslipidemia in 26,378 individuals from rural Hua County, China, 2012–2016

Indicator	Classification	Total n(%) (N = 26,378)	Male n(%) (*N* = 12,925)	Female n(%) (*N* = 13,453)	*P* value ^a^
TC	Ideal	17,930 (67.97)	9374 (72.53)	8556 (63.60)	< 0.001
	Borderline high	6470 (24.53)	2799 (21.66)	3671 (27.29)	
	High	1978 (7.50)	752 (5.82)	1226 (9.11)	
TG	Ideal	18,353 (69.58)	9165 (70.91)	9188 (68.30)	0.004
	Borderline high	4192 (15.89)	1837 (14.21)	2355 (17.51)	
	High	3833 (14.53)	1923 (14.88)	1910 (14.20)	
LDL-C	Ideal	23,854 (90.43)	11,864 (91.79)	11,990 (89.13)	< 0.001
	Borderline high	2059 (7.81)	863 (6.68)	1196 (8.89)	
	High	465 (1.76)	198 (1.53)	267 (1.98)	
HDL-C	Normal	23,955 (90.81)	11,521 (89.14)	12,434 (92.43)	< 0.001
	Low	2423 (9.19)	1404 (10.86)	1019 (7.57)	
BHA dyslipidemia ^b^	Normal	13,538 (51.32)	7146 (55.29)	6392 (47.51)	< 0.001
	Abnormal	12,840 (48.68)	5779 (44.71)	7061 (52.49)	
High dyslipidemia ^c^	Normal	21,129 (80.10)	10,494 (81.19)	10,635 (79.05)	< 0.001
	Abnormal	5249 (19.90)	2431 (18.81)	2818 (20.95)	

^a^*P* values were derived from the Chi-square test or Ridit test

^b^ BHA (borderline high and above) dyslipidemia was defined as presence of borderline high or high level in any one of the factors TC, TG or LDL-C

^c^ High dyslipidemia was defined as presence of high level of any one of TC, TG or LDL-C

As shown in Fig. 1c-e, the age related trend for prevalence of the BHA status of TC, TG and LDL-C demonstrated a “scissor-shaped” cross pattern between genders: the prevalence of BHA TC, TG and LDL-C rose with increasing age in females but remained stable or even decreased in males, resulting in intersection of these two curves in the 50–55-year age group (*P*_interaction_ < 0.001). Similar scissor-shaped patterns were also observed for BHA dyslipidemia and high dyslipidemia as defined in this study (Fig. 1a-b), and high-level groups in TC, TG and LDL-C (Supplementary Figure 2). In contrast, the prevalence of low HDL-C showed no striking increasing or decreasing trend with age in males and females in this study (Fig. 1f, *P*_interaction_ = 0.969).

For factors associated with BHA dyslipidemia, significant heterogeneity in OR estimates of age, BMI and blood pressure was found in males vs. females. The risk of dyslipidemia was associated predominantly with obesity in males, but was more predominantly associated with hypertension in females (Table 3). In view of these differences, multiple logistic regression models were fitted in males and females separately (Table 3). For males, younger age, nonphysical work, higher BMI, hypertension, use of a shallow well as a main source of drinking water, alcohol use, more salty food intake and symptoms of heartburn and regurgitation were associated with an increased risk of BHA dyslipidemia. Risk factors for BHA dyslipidemia in females included older age, nonphysical work, higher BMI, hypertension, use of a shallow well as a main source of drinking water, less intake of fried food, more intake of spicy food, symptoms of heartburn and regurgitation and a self-reported history of diabetes (Table 3). Similar risk factor patterns were observed in models taking high dyslipidemia as a dependent variable (Supplementary Table 4).

**Table 3:** Associated factors for BHA dyslipidemia in stratification of gender identified from 26,378 participants from rural Hua County, China, 2012–2016

Variable	Male (*N* = 12,925)				Female (*N* = 13,453)				*P* value for interaction^c^
	Abnormal / Normal numbers (n1/n2)	Crude OR ^a^ (95%CI)	Adjusted OR ^b^ (95%CI)	β coefficient (SE) ^b^	Abnormal / Normal numbers (n1/n2)	Crude OR ^a^ (95%CI)	Adjusted OR ^b^ (95%CI)	β coefficient (SE) ^b^	
Age group									
45–49	1371/1330	Ref	Ref	–	1198/1947	Ref	Ref	–	< 0.001
50–54	1055/1187	0.86 (0.77–0.96)	0.91 (0.81–1.02)	−0.09 (0.06)	1262/1364	1.50 (1.35–1.67)	1.51 (1.35–1.68)	0.41 (0.05)	
55–59	1187/1503	0.77 (0.69–0.85)	0.88 (0.79–0.99)	− 0.12 (0.06)	1597/1144	2.27 (2.04–2.52)	2.32 (2.08–2.58)	0.84 (0.05)	
60–64	1349/1890	0.69 (0.62–0.77)	0.81 (0.72–0.90)	−0.22 (0.06)	1797/1205	2.42 (2.19–2.69)	2.49 (2.24–2.77)	0.91 (0.05)	
65–69	817/1236	0.64 (0.57–0.72)	0.76 (0.67–0.86)	−0.27 (0.06)	1207/732	2.68 (2.38–3.01)	2.76 (2.45–3.11)	1.02 (0.06)	
*P*_trend_^d^		< 0.001	< 0.001			< 0.001	< 0.001		
Job									
Physical worker	5564/6980	Ref	Ref	–	7010/6355	Ref	Ref	–	0.560
Nonphysical worker	215/166	1.62 (1.32–2.00)	1.38 (1.11–1.71)	0.32 (0.11)	51/37	1.25 (0.82–1.91)	1.57 (1.01–2.44)	0.45 (0.22)	
Body mass index									
≤ 24.0 kg/m^2^	1552/3259	Ref	Ref	–	2232/2765	Ref	Ref	–	< 0.001
24.1–27.9 kg/m^2^	2550/2844	1.88 (1.74–2.04)	1.84 (1.69–1.99)	0.61 (0.04)	2990/2485	1.49 (1.38–1.61)	1.52 (1.40–1.65)	0.42 (0.04)	
≥ 28.0 kg/m^2^	1655/1015	3.42 (3.10–3.78)	3.19 (2.88–3.53)	1.16 (0.05)	1819/1129	2.00 (1.82–2.19)	2.03 (1.84–2.23)	0.71 (0.05)	
*P*_trend_^d^		< 0.001	< 0.001			< 0.001	< 0.001		
Blood pressure									
Nonhypertension	2312/3270	Ref	Ref	–	3066/3371	Ref	Ref	–	0.003
Hypertension	3432/3828	1.27 (1.18–1.36)	1.11 (1.03–1.20)	0.11 (0.04)	3957/2995	1.45 (1.36–1.56)	1.24 (1.16–1.33)	0.22 (0.04)	
Source of drinking water									
Deep well	3734/4819	Ref	Ref	–	4598/4324	Ref	Ref	–	0.735
Shallow well or others	2045/2327	1.13 (1.05–1.22)	1.21 (1.12–1.30)	0.19 (0.04)	2463/2068	1.12 (1.04–1.20)	1.14 (1.06–1.23)	0.13 (0.04)	
Smoking									
No	1773/2195	Ref	Ref	–	6976/6325	Ref	Ref	–	0.708
Moderate amount	3005/3652	1.02 (0.94–1.10)	1.03 (0.95–1.12)	0.03 (0.04)	79/61	1.17 (0.84–1.64)	0.98 (0.69–1.39)	−0.02 (0.18)	
Large amount	985/1270	0.96 (0.87–1.07)	0.97 (0.87–1.09)	− 0.03 (0.06)	5/5	0.91 (0.26–3.13)	0.65 (0.18–2.43)	− 0.43 (0.67)	
*P*_trend_^d^		0.576	0.788			0.452	0.709		
Alcohol drinking									
No	2969/4227	Ref	Ref	–	7017/6356	Ref	Ref	–	0.461
Moderate amount	1992/2285	1.24 (1.15–1.34)	1.16 (1.07–1.25)	0.15 (0.04)	43/31	1.26 (0.79–2.00)	1.26 (0.78–2.04)	0.23 (0.25)	
Large amount	813/628	1.84 (1.64–2.07)	1.65 (1.46–1.87)	0.50 (0.06)	1/3	0.30 (0.03–2.90)	0.24 (0.02–2.52)	−1.42 (1.19)	
*P*_trend_^d^		< 0.001	< 0.001			0.679	0.728		
Fried food intake									
Seldom	3894/4949	Ref	Ref	–	5255/4669	Ref	Ref	–	0.112
Often	1885/2197	1.09 (1.01–1.17)	0.98 (0.90–1.06)	−0.02 (0.04)	1806/1723	0.93 (0.86–1.01)	0.89 (0.82–0.97)	− 0.12 (0.04)	
Salty food intake									
Seldom	940/1244	Ref	Ref	–	1543/1494	Ref	Ref	–	0.816
Often	4839/902	1.09 (0.99–1.19)	1.10 (1.00–1.21)	0.09 (0.05)	5518/4898	0.95 (0.82–1.10)	1.06 (0.97–1.15)	0.06 (0.04)	
Spicy food intake									0.887
Seldom	3489/4604	Ref	Ref	–	4538/4257	Ref	Ref	–	
Often	2290/2542	1.19 (1.11–1.28)	1.05 (0.97–1.14)	0.05 (0.04)	2523/2135	1.11 (1.03–1.19)	1.20 (1.11–1.30)	0.18 (0.04)	
Heartburn and regurgitation									
No	4025/5195	Ref	Ref	–	5130/4843	Ref	Ref	–	0.535
Yes	1754/1951	1.16 (1.07–1.25)	1.08 (1.00–1.17)	0.08 (0.04)	1931/1549	1.18 (1.09–1.27)	1.13 (1.05–1.23)	0.12 (0.04)	
Self-reported diabetes history									
No	5648/7036	Ref	Ref	–	6867/6283	Ref	Ref	–	0.560
Yes	131/110	1.48 (1.15–1.92)	1.30 (0.99–1.69)	0.26 (0.14)	194/109	1.63 (1.28–2.06)	1.31 (1.03–1.67)	0.27 (0.12)	

^a^ Crude OR was calculated from Univariate Logistic Model

^b^ Coefficient and adjusted OR were calculated from Multiple Logistic Model. Adjustment variables included age group, job, BMI, blood pressure, source of drinking water, smoking, alcohol drinking, fried food intake, salty food intake, spicy food intake, heartburn and regurgitation and self-reported diabetes history

^c^*P* value for interaction was derived by adding the interaction term of the specific risk factor (one term at a time) and gender variable into the model

^d^*P* values were derived from the Cochran-Armitage test for trend

## Discussion

Mortality associated with CVD has risen in China over the last decade, and has become the leading cause of mortality and accounts for over 40% of deaths from all causes in 2014 [21, 22]. Blood lipids are a principal contributor to the onset of CVD, and control of these factors has increasingly been a concern of health policy makers in China. Guidelines for prevention and treatment of dyslipidemia in Chinese adults were established in 2007 [2, 3]. The first-phase pilot community-based blood lipid management project was completed in 2013–2016 in urban areas of China (Beijing, Hangzhou and Shenzhen City), and the second-phase of this project is now underway in rural areas [17]. However, the practice of dyslipidemia management is still limited in rural China. Due to the large number of residents, the relatively low level of economic development and limited access to health resources in rural areas, establishment of a population-level blood lipid management project requires great circumspection. This study for the first time systematically evaluated the gender heterogeneity in prevalence of dyslipidemia, trends of dyslipidemia with age, and associated factors based on data from a large-scale population-based study in rural China. Findings from this study have shed light on the potential for a “tailored” strategy of dyslipidemia management.

A significant finding of this study was the heterogeneity regarding trend with age of dyslipidemia in males and females in middle aged rural Chinese. Prevalence of dyslipidemia was significantly higher in females in the whole population under study, but it demonstrates a “scissor shaped” trend with age between genders, which crossed at the age of 50–55 years. Although this gender associated heterogeneity has not been previously well recognized, there have been similar results in population-based surveys in China. Studies by Zhang M et al., Pan L et al. and Qi L et al. [9–11] with representative sampling framework demonstrated that the prevalence of high TC, TG and LDL-C increase with age in females but are stable or decreased in males in subjects of 40–70 years, and showed a “scissor shaped” pattern very similar to that observed in our study population (Supplementary Figure 3A-D & E-H & I). Investigations in Korea [23, 24], Brazil [25] and India [26] have also reported that age trend of abnormal TC, TG or LDL-C showed a “scissor-shaped” pattern between genders. Mechanisms underlying this “scissor shaped” age related trend of prevalence in dyslipidemia between genders are not completely clear. Possible explanations for this trend of increase in females include menopausal transition and loss of estrogen, which might act as a trigger factor and enhance metabolic dysfunction [27–30]. However, considering blood lipid management, greater attention should be given to males aged 45–50 years and females over 50 in both dyslipidemia screening and intervention in rural China, since these subgroups of individuals were at the highest risk of having dyslipidemia in males and females aged 45–69, respectively.

This study identified several well-established contributing factors for dyslipidemia (e.g. nonphysical work, obesity, hypertension) [12, 13, 31] as well as several gender specific factors. Gender-specific risk factors included alcohol consumption and salty food intake in males, and spicy food intake and self-reported diabetes history in females. It is of interest that the association of dyslipidemia with BMI and blood pressure is modified by gender. Risk of dyslipidemia was associated predominantly with obesity in males, but was more predominantly associated with hypertension in females. The gender-specific risk factor patterns may be useful in identification of a target population for dyslipidemia screening or intervention in the lipid management project.

According to findings of this study, it is proposed that gender specific pattern of dyslipidemia should be taken into careful consideration in population-level blood lipid management and CVD prevention projects in rural China, as: 1) The averaged disease burden (overall prevalence) estimated over the whole population may not in fact represent the situation for either males or females. 2) From the point of project implementation, imprecision in targeting a high-risk population may lead to lack of prioritization and impair the effect of the project.

Results from this study showed that prevalence of HDL-C abnormality had a completely different age-related trend as compared to that of TC, TG and LDL-C. The prevalence of low HDL-C was higher in males than that in females with a relatively fixed absolute difference, which showed two parallel curves without striking increasing or decreasing trend with age in both genders. This distinct pattern of HDL-C as compared to TC, TG and LDL-C was also suggested in previous population-based investigations in China [9, 10] (Supplementary Figure 3D & H). Unlike the other three lipid indicators [32, 33], the biologic function of HDL-C is still unclear and the causal relationship between HDL-C and CVD is uncertain [34]. Several randomized trials using drugs which increase HDL-C failed to show a reduction in CVD events or confer a cardiovascular benefit [35–37]. This evidence indicated that HDL-C may have a unique biologic role in the lipid metabolism process. As such, dyslipidemia in this study was defined as the presence of a borderline high or high level of any one of TC, TG or LDL-C, not including HDL-C. It was different from other studies in which dyslipidemia was defined on the basis of four lipid indicators [10–12, 15].

### Study strengths and limitations

This study is conducted based on a population-based randomized controlled trial with strict quality control, which ensures a good representation of the study population and the reliability of data collected. This study also has limitations. First, this study enrolled only adults aged 45–69, and did not evaluate heterogeneity in prevalence of dyslipidemia in younger individuals. Second, ESECC participants accounted for about 20% of all eligible residents in target villages. Although the age and gender distributions of participants in this study are comparable with those in the entire population of Hua County (data not shown), potential selection bias cannot be ruled out. Third, this was a single-center cross-sectional study, and further population-based multi-center studies would be required to confirm the heterogeneity of dyslipidemia which was identified. Lastly, the questionnaire used in this study was not designated as a lipid specialized survey tool, and several dyslipidemia related risk factors such as intake of fat, intake of sugar, or physical activity were not included.

## Conclusion

Gender heterogeneity was detected in the prevalence, age related trends and risk factor patterns of dyslipidemia in middle aged rural Chinese. Gender-specific estimates should be provided in future lipid prevalence surveys. This heterogeneity should be taken into consideration in population-level blood lipid management projects. The effectiveness of population-level blood lipid management and CVD primary prevention programs in China is expected to be improved if gender heterogeneity is considered.

## Supplementary information

**Additional file 1: Supplementary Table 1.** Definitions and coding forms for potential risk factors investigated in the ESECC trial from rural Hua County, China, 2012–2016. **Supplementary Table 2.** Selected demographic and behavioral characteristics in individuals enrolled in and excluded from the current study from the ESECC trial. **Supplementary Table 3.** Age and gender specific mean level (mg/dL) and prevalence of dyslipidemia among 26,378 participants from rural Hua County, China, 2012–2016. **Supplementary Table 4.** Associated factors for high dyslipidemia identified in stratification of gender from 26,378 participants from rural Hua County, China, 2012–2016. **Supplementary Figure 1.** Flowchart of participant enrollment in this study. **Supplementary Figure 2.** The age and gender distribution of prevalence of high TG, TC and LDL-C among 26,378 individuals from rural Hua County, China, 2012–2016. **Supplementary Figure 3.** The prevalence of high TC, high TG, high LDL-C and low HDL-C in individuals 40–69 years in the 2013–2014 China Chronic Disease and Risk Factor Surveillance (CCDRFS), The China National Survey of Chronic Kidney Disease (CKD) and dyslipidemia investigation in Chongqing.

## Data Availability

The datasets used and/or analysed during the current study are available from the corresponding author on reasonable request.

## Abbreviations

BHA: Borderline high and above
CVD: Cardiovascular disease
HDL-C: High-density lipoprotein cholesterol
LDL-C: Low-density lipoprotein cholesterol
TC: Total cholesterol
TG: Triglycerides
