# Supplementary material for: Gender heterogeneity in dyslipidemia prevalence, trends with age and associated factors in middle age rural Chinese
Source: Lipids Health Dis. 2020 Jun 12;19:135. doi: 10.1186/s12944-020-01313-8 (PMC7291723; doi:10.1186/s12944-020-01313-8)
Supplement: Supplementary file 1 — Additional file 1: Supplementary Table 1. Definitions and coding forms for potential risk factors investigated in the ESECC trial from rural Hua County, China, 2012–2016. Supplementary Table 2. Selected demographic and behavioral characteristics in individuals enrolled in and excluded from the current study from the ESECC trial. Supplementary Table 3. Age and gender specific mean level (mg/dL) and prevalence of dyslipidemia among 26,378 participants from rural Hua County, China, 2012–2016. Supplementary Table 4. Associated factors for high dyslipidemia identified in stratification of gender from 26,378 participants from rural Hua County, China, 2012–2016. Supplementary Figure 1. Flowchart of participant enrollment in this study. Supplementary Figure 2. The age and gender distribution of prevalence of high TG, TC and LDL-C among 26,378 individuals from rural Hua County, China, 2012–2016. Supplementary Figure 3. The prevalence of high TC, high TG, high LDL-C and low HDL-C in individuals 40–69 years in the 2013–2014 China Chronic Disease and Risk Factor Surveillance (CCDRFS), The China National Survey of Chronic Kidney Disease (CKD) and dyslipidemia investigation in Chongqing. [file 12944_2020_1313_MOESM1_ESM.docx]

**SUPPLEMENTAL MATERIAL**

| **Supplementary Table 1. Definitions and coding forms for potential risk factors investigated in the ESECC trial from rural Hua County, China, 2012-2016.** | |
| --- | --- |
| Variable | Definition |
| Gender | Participants were asked their gender. Male was coded as 0 and female coded as 1. |
| Age | Age at enrollment was calculated as Round ((Interview Date-Birthday Date)/365.25). Age groups were categorized by 5-year intervals. Subjects aged 45-49 were coded as 0, subjects aged 50-54 were coded as 1, subjects aged 55-59 were coded as 2, subjects aged 60-64 were coded as 3 and subjects aged 65-69 were coded as 4. |
| Family size | Participants were asked the number of family members living together (including the participant). Family size groups were categorized by number of family members. Subjects living alone were coded as 0, living with 2-4 family members together were coded as 1 and subjects living with more than 5 family members were coded as 2. |
| Educational level | Participants were asked about their educational level in the questionnaire. This question had five options: 1) illiteracy; 2) primary school; 3) middle school; 4) high school; 5) college and above. Subjects selecting 1) were coded as 0, subjects selecting 2) and 3) were coded as 1 and subjects selecting 4) and 5) were coded as 2. |
| Income group | Participants were asked the number of family members living together (including the participant) and the annual income of the entire family. Annual income per capita was calculated as total family income divided by number of family members. Income groups were categorized in quartiles. Subjects with annual income per capita ≥100 Yuan and income < 1500 Yuan were coded as 0; subjects with annual income per capita ≥1500 Yuan and income < 2500 Yuan were coded as 1; subjects with annual income per capita ≥2500 Yuan and income < 5000 Yuan were coded as 2; subjects with annual income per capita ≥5000 Yuan and income < 30000 Yuan were coded as 3. |
| Marital status | Participants were asked about their marital status in the questionnaire. This question had four options: 1) single; 2) married; 3) divorced; 4) widowed. Subjects selecting 2) were coded as 0 and subjects selecting 1), 3) or 4) were coded as 1. |
| Job | Participants were asked about their occupation in the questionnaire. This question had five options: 1) manager; 2) office staff; 3) technician; 4) manual labor; 5) farmer or others. Subjects selecting 1), 2) or 3) were defined as a nonphysical worker and coded as 0; and subjects selecting 4) or 5) were defined as a physical worker and coded as 1. |
| Body mass index | BMI was calculated as body weight in kilograms divided by the square of body height in meters (kg/m^2^). Subjects were categorized into three groups as BMI ≤ 24 (normal, coded as 0), 24<BMI<28 (overweight, coded as 1) and BMI ≥28 (obesity, coded as 2). |
| Blood pressure | Blood pressure was measured for each participant. Subjects with systolic blood pressure ≥ 140 mmHg or diastolic blood pressure ≥ 90 mmHg were defined as hypertensive and coded as 1; others were defined as non-hypertensive and coded as 0. |
| Source of drinking water | Participants were asked about their drinking water source in the questionnaire. This question had two options: 1) deep well (>100 meters); 2) shallow well or others (≤100 meters). Subjects selecting 1) were coded as 0, and subjects selecting 2) were coded as 1. |
| Variable | Definition |
| Cooking frequency | Participants were asked about the frequency of personally doing cooking. This question had three options: 1) seldom (<1 time a week); 2) occasionally (1-3 times a week); 3) often (≥4 times a week). Subjects selecting 1) were coded as 0; and subjects selecting 2) or 3) were coded as 1. |
| Smoking | Participants were asked whether they smoked, if so over what period of time, and quantity of smoking in the questionnaire. Total amounts of cigarette consumption were calculated as time period multiplied by quantity of smoking. The smoking group was categorized by quartiles of accumulative consumption. Subjects who didn't smoke were coded as 0; subjects with consumption in Q1-Q3 were defined as moderate-smokers and coded as 1; subjects with consumption in Q4 were defined as heavy-smokers and coded as 2. |
| Alcohol consumption | Participants were asked whether they had drunk alcohol and if so over what period of time and in what quantity in the questionnaire. Total amounts of alcohol consumption were calculated as period of time multiplied by quantity of drinking. The alcohol drinking groups were categorized by quartiles of accumulative consumption. Subjects who didn't drink were coded as 0; subjects with consumption in Q1-Q3 were defined as moderate drinkers and coded as 1; subjects with consumption in Q4 were defined as heavy drinkers and coded as 2. |
| Fruit and vegetable intake | Participants were asked about their frequency of fruit and vegetables intake separately in the questionnaire. These questions had three options: 1) seldom (<1 time a week); 2) occasionally (1-3 times a week); 3) often (≥4 times a week). Subjects with infrequent fruit intake and infrequent vegetable intake were coded as 0, and other responses were coded as 1. |
| Protein intake | Participants were asked about the frequency of protein intake in the questionnaire. This question had three options: 1) seldom (<1 time a week); 2) occasionally (1-3 times a week); 3) often (≥4 times a week). Subjects selecting 1) were coded as 0 and subjects selecting 2) or 3) were coded as 1. |
| Fried food intake | Participants were asked about the frequency of fried food intake in the questionnaire. This question had three options: 1) seldom (<1 time a week); 2) occasionally (1-3 times a week); 3) often (≥4 times a week). Subjects selecting 1) were coded as 0 and subjects selecting 2) or 3) were coded as 1. |
| Salty food intake | Participants were asked about the frequency of salty food intake in the questionnaire. This question had three options: 1) seldom (<1 time a week); 2) occasionally (1-3 times a week); 3) often (≥4 times a week). Subjects selecting 1) were coded as 0 and subjects selecting 2) or 3) were coded as 1. |
| Spicy food intake | Participants were asked about the frequency of spicy food intake in the questionnaire. This question had three options: 1) seldom (<1 time a week); 2) occasionally (1-3 times a week); 3) often (≥4 times a week). Subjects selecting 1) were coded as 0 and subjects selecting 2) or 3) were coded as 1. |
| Heartburn and regurgitation | Participants were asked whether they had symptoms of heartburn and regurgitation in the questionnaire. This question had two options: 1) no; 2) yes. Subjects selecting 1) were coded as 0 and subjects selecting 2) were coded as 1. |
| Self-reported CVD history | Participants were asked about their medical history in the questionnaire. Subjects reporting a history of CVD were coded as 1 and others were coded as 0. |
| Variable | Definition |
| Self-reported diabetes history | Participants were asked about their medical history in the questionnaire. Subjects reporting a history of diabetes were coded as 1 and others were coded as 0. |

| **Supplementary Table 2. Selected demographic and behavioral characteristics in individuals enrolled in and excluded from the current study from the ESECC trial.** | | | | | | |
| --- | --- | --- | --- | --- | --- | --- |
| Variable | | ESECC participants with informed consent (N=35772) n(%) | Participants enrolled in the current study (N=26378) n(%) | Participants excluded from the current study (N=9394) n(%) | *P* value ^a^ | |
| Age | |  |  |  |  | |
| Mean (SD) | | 56.57 (6.90) | 56.52 (6.88) | 56.73 (6.95) | 0.010 | |
| Gender | |  |  |  |  | |
| Male | | 17468 (48.83) | 12925 (49.00) | 4543 (48.36) | 0.288 | |
| Female | | 18304 (51.17) | 13453 (51.00) | 4851 (51.64) |  | |
| Educational level ^b^ | |  |  |  |  | |
| Illiterate | | 10420 (31.15) | 8537 (32.36) | 1883 (26.63) | <0.001 | |
| Primary or Middle School | | 19693 (58.87) | 15252 (57.82) | 4441 (62.81) |  | |
| High school or above | | 3336 (9.97) | 2589 (9.81) | 747 (10.56) |  | |
| Job ^b^ | |  |  |  |  | |
| Physical worker | | 32846 (98.20) | 25909 (98.22) | 6937 (98.10) | 0.511 | |
| Nonphysical worker | | 603 (1.80) | 469 (1.78) | 134 (1.90) |  | |
| Body mass index | |  |  |  |  | |
| ≤24.0 kg/m^2^ | | 12860 (35.95) | 9808 (37.18) | 3052 (32.49) | <0.001 | |
| -24.1-27.9 kg/m^2^ | | 14905 (41.67) | 10869 (41.20) | 4036 (42.96) |  | |
| ≥28.0 kg/m^2^ | | 7896 (22.07) | 5618 (21.30) | 2278 (24.25) |  | |
| Unknown ^c^ | | 111 (0.31) | 83 (0.31) | 28 (0.30) |  | |
| Blood pressure | |  |  |  |  | |
| No hypertension | | 15712 (43.92) | 12019 (45.56) | 3693 (39.31) | <0.001 | |
| Hypertension | | 19859 (55.52) | 14212 (53.88) | 5647 (60.11) |  | |
| Unknown ^c^ | | 201 (0.56) | 147 (0.56) | 54 (0.57) |  | |
| Water source ^b^ | |  |  |  |  | |
| Deep well | | 23569 (70.46) | 17475 (66.25) | 6094 (86.18) | <0.001 | |
| Shallow well or other | | 9880 (29.54) | 8903 (33.75) | 977 (13.82) | |  |
| Variable | | ESECC participants with informed consent (N=35772) n(%) | Participants enrolled in the current study (N=26378) n(%) | Participants excluded from the current study (N=9394) n(%) | | *p* value^a^ |
| Smoking ^b^ |  | |  |  | |  |
| None | 21846 (65.31) | | 17269 (65.47) | 4577 (64.72) | | 0.451 |
| Moderate amount | 8667 (25.91) | | 6796 (25.76) | 1871 (26.46) | |  |
| Large amount | 2872 (8.59) | | 2265 (8.59) | 607 (8.58) | |  |
| Unknown ^c^ | 64 (0.19) | | 47 (0.18) | 2339 (0.24) | |  |
| Alcohol consumption ^b^ |  | |  |  | |  |
| None | 26097 (78.02) | | 20568 (77.97) | 5529 (78.19) | | 0.254 |
| Moderate amount | 5474 (16.37) | | 4351 (16.49) | 1123 (15.88) | |  |
| Large amount | 1859 (5.56) | | 1445 (5.48) | 414 (5.85) | |  |
| Unknown ^c^ | 19 (0.06) | | 14 (0.05) | 5 (0.07) | |  |
| Fried food intake ^b^ |  | |  |  | |  |
| Seldom | 23951 (71.60) | | 18767 (71.15) | 5184 (73.31) | | <0.001 |
| Often | 9498 (28.40) | | 7611 (28.85) | 1887 (26.69) | |  |
| Spicy food intake ^b^ |  | |  |  | |  |
| Seldom | 21609 (64.60) | | 16888 (64.02) | 4721 (66.77) | | <0.001 |
| Often | 11840 (35.40) | | 9490 (35.98) | 2350 (33.23) | |  |
| Heartburn and regurgitation ^b^ |  | |  |  | |  |
| No | 24006 (71.77) | | 19193 (72.76) | 4813 (68.07) | | <0.001 |
| Yes | 9443 (28.23) | | 7185 (27.24) | 2258 (31.93) | |  |
| Self-reported history of diabetes ^b^ |  | |  |  | |  |
| No | 32695 (97.75) | | 25834 (97.94) | 6861 (97.03) | | <0.001 |
| Yes | 754 (2.25) | | 544 (2.06) | 210 (2.97) | |  |
| ^a^ The Chi-square test and Student's t test were used to compare demographic characteristics and behavioral factors among individuals enrolled in and not enrolled in the ESECC, as well as male and female subjects included in this study. | | | | | | |
| ^b^ There were 2323 participants who did not complete the questionnaire investigation not shown in the table. | | | | | | |
| ^c^ The "Unknown" category was not included in the analysis. | | | | | | |

| **Supplementary Table 3. Age and gender specific mean level (mg/dL) and prevalence of dyslipidemia among 26378 participants from rural Hua County, China, 2012-2016.** | | | | | | | | | | | | | | | | | | | | | | | | |
| --- | --- | --- | --- | --- | --- | --- | --- | --- | --- | --- | --- | --- | --- | --- | --- | --- | --- | --- | --- | --- | --- | --- | --- | --- |
| Age (year) | TC | | | |  | TG | | | |  | LDL-C | | | |  | HDL-C | | |  | BHA dyslipidemia ^a^ | |  | High Dyslipidemia ^b^ | |
|  | Mean (SD) | Ideal n(%) | Borderline High n(%) | High n(%) |  | Mean (SD) | Ideal n(%) | Borderline High n(%) | High n(%) |  | Mean (SD) | Ideal n(%) | Borderline High n(%) | High n(%) |  | Mean (SD) | Normal n(%) | Low n(%) |  | Normal n(%) | Abnormal n(%) |  | Normal n(%) | Abnormal n(%) |
| Total (N=26378) | |  |  |  |  |  |  |  |  |  |  |  |  |  |  |  |  |  |  |  |  |  |  |  |
| 45-49 | 180.02 (34.26) | 4402 (75.30) | 1151 (19.69) | 293 (5.01) |  | 144.01 (141.93) | 4078 (69.76) | 860 (14.71) | 908 (15.53) |  | 93.99 (24.51) | 5443 (93.11) | 335 (5.73) | 68 (1.16) |  | 52.28 (13.99) | 5266 (90.08) | 580 (9.92) |  | 3277 (56.06) | 2569 (43.94) |  | 4739 (81.06) | 1107 (18.94) |
| 50-54 | 184.08 (35.08) | 3442 (70.71) | 1099 (22.58) | 327 (6.72) |  | 141.65 (109.93) | 3330 (68.41) | 801 (16.45) | 737 (15.14) |  | 97.05 (24.51) | 4446 (91.33) | 347 (7.13) | 75 (1.54) |  | 51.11 (13.57) | 4340 (89.15) | 528 (10.85) |  | 2551 (52.40) | 2317 (47.60) |  | 3903 (80.18) | 965 (19.82) |
| 55-59 | 189.51 (35.30) | 3537 (65.13) | 1453 (26.75) | 441 (8.12) |  | 140.55 (102.57) | 3716 (68.42) | 881 (16.22) | 834 (15.36) |  | 98.39 (25.73) | 4884 (89.93) | 434 (7.99) | 113 (2.08) |  | 54.24 (14.77) | 4984 (91.77) | 447 (8.23) |  | 2647 (48.74) | 2784 (51.26) |  | 4291 (79.01) | 1140 (20.99) |
| 60-64 | 189.39 (36.08) | 4002 (64.12) | 1706 (27.34) | 533 (8.54) |  | 135.93 (97.79) | 4405 (70.58) | 983 (15.75) | 853 (13.67) |  | 99.48 (25.35) | 5573 (89.30) | 549 (8.80) | 119 (1.90) |  | 53.05 (13.95) | 5712 (91.52) | 529 (8.48) |  | 3095 (49.59) | 3146 (50.41) |  | 5004 (80.18) | 1237 (19.82) |
| 65-69 | 190.22 (36.81) | 2547 (63.80) | 1061 (26.58) | 384 (9.62) |  | 134.15 (95.45) | 2824 (70.74) | 667 (16.71) | 501 (12.55) |  | 100.04 (25.97) | 3508 (87.88) | 394 (9.87) | 90 (2.25) |  | 52.87 (13.80) | 3653 (91.51) | 339 (8.49) |  | 1968 (49.30) | 2024 (50.70) |  | 3192 (79.96) | 800 (20.04) |
| Total | 186.48 (35.68) | 17930 (67.97) | 6470 (24.53) | 1978 (7.50) |  | 139.46 (111.86) | 18353 (69.58) | 4192 (15.89) | 3833 (14.53) |  | 97.67 (25.28) | 23854 (90.43) | 2059 (7.81) | 465 (1.76) |  | 52.74 (14.08) | 23955 (90.81) | 2423 (9.19) |  | 13538 (51.32) | 12840 (48.68) | | 21129 (80.10) | 5249 (19.90) |
| Male (N=12925) | |  |  |  |  |  |  |  |  |  |  |  |  |  |  |  |  |  |  |  |  |  |  |  |
| 45-49 | 182.97 (36.17) | 1936 (71.68) | 590 (21.84) | 175 (6.48) |  | 166.02 (180.88) | 1705 (63.12) | 401 (14.85) | 595 (22.03) |  | 95.81 (25.63) | 2465 (91.26) | 189 (7.00) | 47 (1.74) |  | 51.36 (13.20) | 2376 (87.97) | 325 (12.03) |  | 1330 (49.24) | 1371 (50.76) |  | 1997 (73.94) | 704 (26.06) |
| 50-54 | 181.42 (35.57) | 1646 (73.42) | 449 (20.03) | 147 (6.56) |  | 150.67 (137.62) | 1487 (66.32) | 358 (15.97) | 397 (17.71) |  | 95.88 (24.99) | 2056 (91.70) | 150 (6.69) | 36 (1.61) |  | 50.39 (14.99) | 1953 (87.11) | 289 (12.89) |  | 1187 (52.94) | 1055 (47.06) |  | 1756 (78.32) | 486 (21.68) |
| 55-59 | 182.99 (33.79) | 1958 (72.79) | 584 (21.71) | 148 (5.50) |  | 136.02 (116.63) | 1943 (72.23) | 362 (13.46) | 385 (14.31) |  | 95.43 (24.80) | 2469 (91.78) | 182 (6.77) | 39 (1.45) |  | 53.39 (15.34) | 2435 (90.52) | 255 (9.48) |  | 1503 (55.87) | 1187 (44.13) |  | 2211 (82.19) | 479 (17.81) |
| 60-64 | 182.37 (34.63) | 2328 (71.87) | 736 (22.72) | 175 (5.40) |  | 126.68 (92.97) | 2446 (75.52) | 431 (13.31) | 362 (11.18) |  | 96.35 (24.51) | 2973 (91.79) | 223 (6.88) | 43 (1.33) |  | 52.12 (13.22) | 2900 (89.53) | 339 (10.47) |  | 1890 (58.35) | 1349 (41.65) |  | 2755 (85.06) | 484 (14.94) |
| 65-69 | 181.07 (34.34) | 1506 (73.36) | 440 (21.43) | 107 (5.21) |  | 121.54 (94.25) | 1584 (77.16) | 285 (13.88) | 184 (8.96) |  | 96.34 (24.25) | 1901 (92.60) | 119 (5.80) | 33 (1.61) |  | 52.13 (14.65) | 1857 (90.45) | 196 (9.55) |  | 1236 (60.20) | 817 (39.80) |  | 1775 (86.46) | 278 (13.54) |
| Total | 182.25 (34.90) | 9374 (72.53) | 2799 (21.66) | 752 (5.82) |  | 140.19 (129.57) | 9165 (70.91) | 1837 (14.21) | 1923 (14.88) |  | 95.96 (24.85) | 11864 (91.79) | 863 (6.68) | 198 (1.53) |  | 51.93 (14.25) | 11521 (89.14) | 1404 (10.86) |  | 7146 (55.29) | 5779 (44.71) |  | 10494 (81.19) | 2431 (18.81) |
| Female (N=13453) | |  |  |  |  |  |  |  |  |  |  |  |  |  |  |  |  |  |  |  |  |  |  |  |
| 45-49 | 177.48 (32.32) | 2466 (78.41) | 561 (17.84) | 118 (3.75) |  | 125.10 (92.62) | 2373 (75.45) | 459 (14.59) | 313 (9.95) |  | 92.42 (23.40) | 2978 (94.69) | 146 (4.64) | 21 (0.67) |  | 53.07 (14.59) | 2890 (91.89) | 255 (8.11) |  | 1947 (61.91) | 1198 (38.09) |  | 2742 (87.19) | 403 (12.81) |
| 50-54 | 186.34 (34.50) | 1796 (68.39) | 650 (24.75) | 180 (6.85) |  | 133.95 (78.16) | 1843 (70.18) | 443 (16.87) | 340 (12.95) |  | 98.06 (24.04) | 2390 (91.01) | 197 (7.5) | 39 (1.49) |  | 51.73 (12.18) | 2387 (90.90) | 239 (9.10) |  | 1364 (51.94) | 1262 (48.06) |  | 2147 (81.76) | 479 (18.24) |
| 55-59 | 195.91 (35.58) | 1579 (57.61) | 869 (31.70) | 293 (10.69) |  | 144.98 (86.37) | 1773 (64.68) | 519 (18.93) | 449 (16.38) |  | 101.28 (26.30) | 2415 (88.11) | 252 (9.19) | 74 (2.70) |  | 55.06 (14.14) | 2549 (93.00) | 192 (7.00) |  | 1144 (41.74) | 1597 (58.26) |  | 2080 (75.88) | 661 (24.12) |
| 60-64 | 196.97 (36.09) | 1674 (55.76) | 970 (32.31) | 358 (11.93) |  | 145.90 (101.80) | 1959 (65.26) | 552 (18.39) | 491 (16.36) |  | 102.87 (25.80) | 2600 (86.61) | 326 (10.86) | 76 (2.53) |  | 54.05 (14.64) | 2812 (93.67) | 190 (6.33) |  | 1205 (40.14) | 1797 (59.86) |  | 2249 (74.92) | 753 (25.08) |
| 65-69 | 199.91 (36.86) | 1041 (53.69) | 621 (32.03) | 277 (14.29) |  | 147.51 (94.93) | 1240 (63.95) | 382 (19.70) | 317 (16.35) |  | 103.95 (27.14) | 1607 (82.88) | 275 (14.18) | 57 (2.94) |  | 53.65 (12.80) | 1796 (92.63) | 143 (7.37) |  | 732 (37.75) | 1207 (62.25) |  | 1417 (73.08) | 522 (26.92) |
| Total | 190.55 (35.94) | 8556 (63.60) | 3671 (27.29) | 1226 (9.11) |  | 138.75 (91.68) | 9188 (68.30) | 2355 (17.50) | 1910 (14.20) |  | 99.32 (25.58) | 11990 (89.13) | 1196 (8.89) | 267 (1.98) |  | 53.52 (13.86) | 12434 (92.43) | 1019 (7.57) |  | 6392 (47.51) | 7061 (52.49) |  | 10635 (79.05) | 2818 (20.95) |
| ^a^ BHA (borderline high and above) dyslipidemia was defined as presence of borderline high or high level of any one of TC, TG or LDL-C. | | | | | | | | | | | | | | | | | | | | | | | | |
| ^b^ High Dyslipidemia was defined as presence of high level of any one of TC, TG or LDL-C. | | | | | | | | | | | | | | | | | | | | | | | | |

| **Supplementary Table 4. Associated factors for high dyslipidemia identified in stratification of gender from 26378 participants from rural Hua County, China, 2012-2016** | | | | | | | | | | |
| --- | --- | --- | --- | --- | --- | --- | --- | --- | --- | --- |
| Variable | Male (N=12925) | | | |  | Female (N=13453) | | | | *P* value for interaction^c^ |
|  | Abnormal cases (n1) | Normal numbers (n2) | Crude OR ^a^ (95%CI) | Adjusted OR ^b^ (95%CI) |  | Abnormal cases (n1) | Normal numbers (n2) | Crude OR ^a^ (95%CI) | Adjusted OR ^b^ (95%CI) |  |
| Age group |  |  |  |  |  |  |  |  |  |  |
| 45-49 | 704 | 1997 | Ref | Ref |  | 403 | 2742 | Ref | Ref | <0.001 |
| 50-54 | 486 | 1756 | 0.79 (0.69-0.9) | 0.84 (0.73-0.96) |  | 479 | 2147 | 1.52 (1.31-1.75) | 1.52 (1.31-1.76) |  |
| 55-59 | 479 | 2211 | 0.61 (0.54-0.7) | 0.72 (0.63-0.82) |  | 661 | 2080 | 2.16 (1.89-2.48) | 2.2 (1.92-2.53) |  |
| 60-64 | 484 | 2755 | 0.5 (0.44-0.57) | 0.58 (0.51-0.67) |  | 753 | 2249 | 2.28 (1.99-2.60) | 2.34 (2.04-2.69) |  |
| 65-69 | 278 | 1775 | 0.44 (0.38-0.52) | 0.53 (0.45-0.62) |  | 522 | 1417 | 2.51 (2.17-2.90) | 2.58 (2.22-3.00) |  |
| *P*_trend_ ^d^ |  |  | <0.001 | <0.001 |  |  |  | <0.001 | <0.001 |  |
| Job |  |  |  |  |  |  |  |  |  |  |
| Physical worker | 2325 | 10219 | Ref | Ref |  | 2795 | 10570 | Ref | Ref | 0.686 |
| Nonphysical worker | 106 | 275 | 1.69 (1.35-2.13) | 1.34 (1.06-1.71) |  | 23 | 65 | 1.34 (0.83-2.16) | 1.63 (1.00-2.67) |  |
| Body mass index |  |  |  |  |  |  |  |  |  |  |
| ≤24.0 kg/m^2^ | 468 | 4343 | Ref | Ref |  | 775 | 4222 | Ref | Ref | <0.001 |
| 24.1-27.9 kg/m^2^ | 1083 | 4311 | 2.33 (2.08-2.62) | 2.21 (1.96-2.49) |  | 1208 | 4267 | 1.54 (1.40-1.70) | 1.56 (1.41-1.72) |  |
| ≥28.0 kg/m^2^ | 871 | 1799 | 4.49 (3.96-5.09) | 3.95 (3.47-4.5) |  | 828 | 2120 | 2.13 (1.90-2.38) | 2.13 (1.90-2.39) |  |
| *P*_trend_ ^d^ |  |  | <0.001 | <0.001 |  |  |  | <0.001 | <0.001 |  |
| Blood pressure |  |  |  |  |  |  |  |  |  |  |
| Nonhypertension | 909 | 4673 | Ref | Ref |  | 1145 | 5292 | Ref | Ref | 0.114 |
| Hypertension | 1508 | 5752 | 1.35 (1.23-1.48) | 1.17 (1.06-1.29) |  | 1655 | 5297 | 1.44 (1.33-1.57) | 1.24 (1.14-1.35) |  |
| Source of drinking water |  |  |  |  |  |  |  |  |  |  |
| Deep well | 1552 | 7001 | Ref | Ref |  | 1795 | 7127 | Ref | Ref | 0.727 |
| Shallow well or others | 879 | 3493 | 1.14 (1.04-1.24) | 1.21 (1.10-1.34) |  | 1023 | 3508 | 1.16 (1.06-1.26) | 1.20 (1.09-1.31) |  |
| Smoking |  |  |  |  |  |  |  |  |  |  |
| No | 733 | 3235 | Ref | Ref |  | 2786 | 10515 | Ref | Ref | 0.439 |
| Moderate amount | 1267 | 5390 | 1.04 (0.94-1.15) | 1.05 (0.94-1.17) |  | 31 | 109 | 1.07 (0.72-1.60) | 0.92 (0.61-1.39) |  |
| Large amount | 423 | 1832 | 1.02 (0.89-1.16) | 1.10 (0.95-1.28) |  | 0 | 10 | - | - |  |
| *P*_trend_ ^d^ |  |  | 0.686 | 0.177 |  |  |  | - | - |  |
| Alcohol drinking |  |  |  |  |  |  |  |  |  |  |
| No | 1158 | 6038 | Ref | Ref |  | 2804 | 10569 | Ref | Ref | 0.056 |
| Moderate amount | 895 | 3382 | 1.38 (1.25-1.52) | 1.23 (1.11-1.36) |  | 14 | 60 | 0.88 (0.49-1.58) | 0.88 (0.48-1.60) |  |
| Large amount | 376 | 1065 | 1.84 (1.61-2.10) | 1.52 (1.31-1.76) |  | 0 | 4 | - | - |  |
| *P*_trend_ ^d^ |  |  | <0.001 | <0.001 |  |  |  | - | - |  |
| Fried food intake |  |  |  |  |  |  |  |  |  |  |
| Seldom | 1627 | 7216 | Ref | Ref |  | 2116 | 7808 | Ref | Ref | 0.179 |
| Often | 804 | 3278 | 1.09 (0.99-1.20) | 0.93 (0.84-1.03) |  | 702 | 2827 | 0.92 (0.83-1.01) | 0.88 (0.79-0.97) |  |
| Salty food intake |  |  |  |  |  |  |  |  |  |  |
| Seldom | 368 | 1816 | Ref | Ref |  | 627 | 2410 | Ref | Ref | 0.051 |
| Often | 2063 | 8678 | 1.17 (1.04-1.33) | 1.19 (1.05-1.36) |  | 2191 | 8225 | 1.02 (0.93-1.13) | 0.99 (0.89-1.09) |  |
| Spicy food intake |  |  |  |  |  |  |  |  |  |  |
| Seldom | 1393 | 6700 | Ref | Ref |  | 1787 | 7008 | Ref | Ref | 0.182 |
| Often | 1038 | 3794 | 1.32 (1.20-1.44) | 1.13 (1.02-1.25) |  | 1031 | 3627 | 1.11 (1.02-1.22) | 1.20 (1.09-1.32) |  |
| Heartburn and regurgitation | |  |  |  |  |  |  |  |  |  |
| No | 1658 | 7562 | Ref | Ref |  | 2012 | 7961 | Ref | Ref | 0.796 |
| Yes | 773 | 2932 | 1.20 (1.09-1.32) | 1.10 (1.00-1.22) |  | 806 | 2674 | 1.19 (1.09-1.31) | 1.14 (1.04-1.26) |  |
| Self-reported diabetes history | |  |  |  |  |  |  |  |  |  |
| No | 2369 | 10315 | Ref | Ref |  | 2716 | 10434 | Ref | Ref | 0.107 |
| Yes | 62 | 179 | 1.51 (1.13-2.02) | 1.32 (0.97-1.79) |  | 102 | 201 | 1.95 (1.53-2.48) | 1.63 (1.27-2.08) |  |
| ^a^ Crude OR was calculated from Univariate Logistic Model | | | | | | | | | | |
| ^b^ Coefficient and adjusted OR were calculated from Multiple Logistic Model. Adjustment variables included age group, job, BMI, blood pressure, source of drinking water, smoking, alcohol drinking, fried food intake, salty food intake, spicy food intake, heartburn and regurgitation and self-reported diabetes history. | | | | | | | | | | |
| ^c^ *P* value for interaction was derived by adding the interaction term of the specific risk factor (one term at a time) and gender variable into the model.  ^d^ *P* values were derived from the Cochran-Armitage test for trend. | | | | | | | | | | |

**
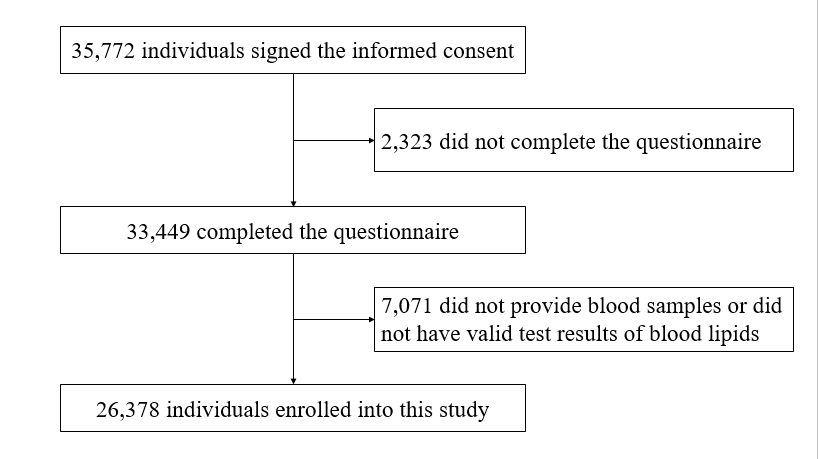
**

**Supplementary Figure 1. Flowchart of participant enrollment in this study.**

**
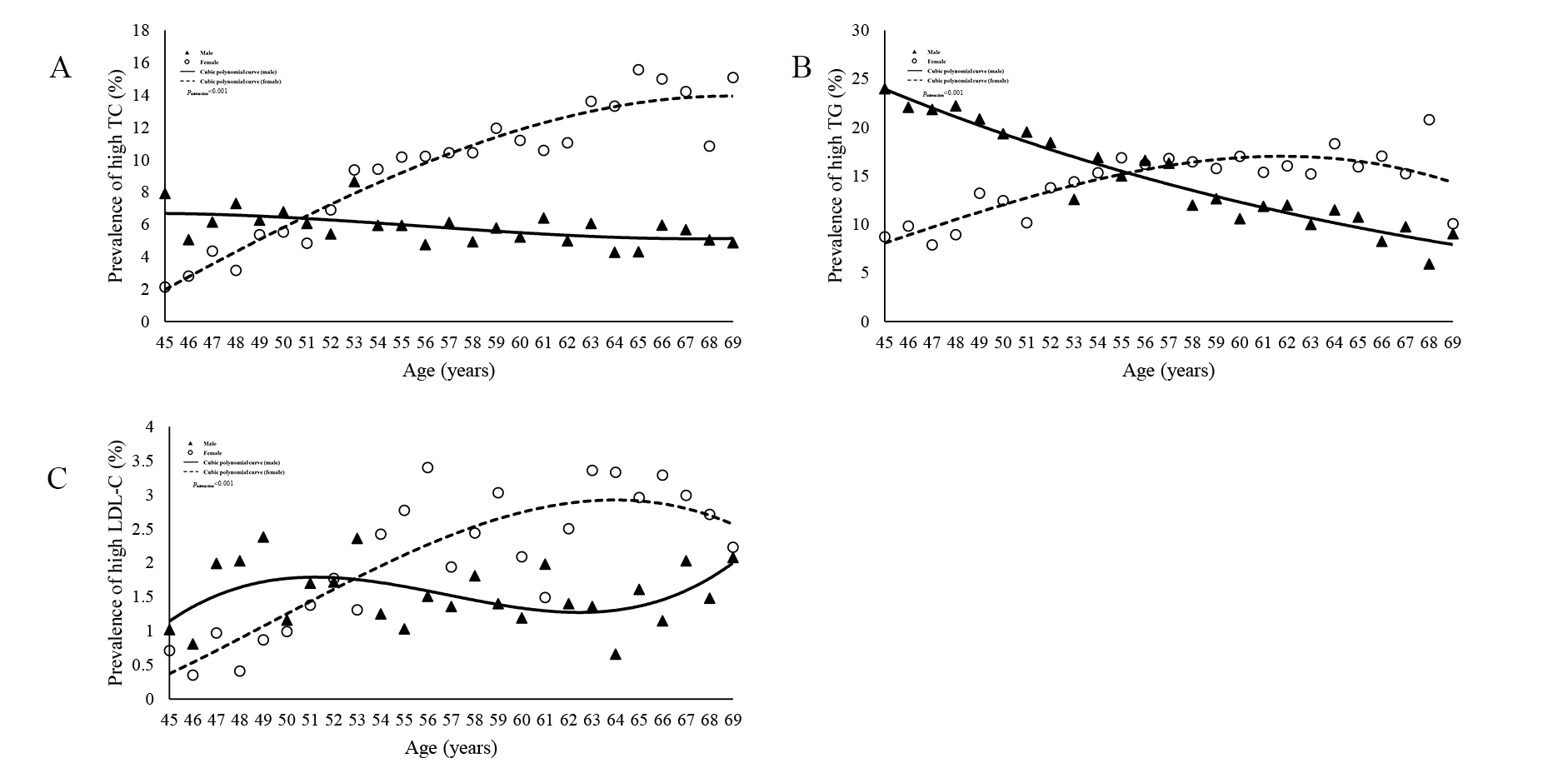
**

**Supplementary Figure 2. The age and gender distribution of prevalence of high TG, TC and LDL-C among 26378 individuals from rural Hua County, China, 2012-2016 ^a^**

A: Age and gender distribution of high total cholesterol (TC).

B: Age and gender distribution of high triglycerides (TG).

C: Age and gender distribution of high low-density lipoprotein cholesterol (LDL-C).

^a^ A cubic polynomial curve was fitted to reduce random fluctuations. Heterogeneity between genders was tested using models with main effects and interaction terms of gender variable and the linear, quadratic and cubic forms of age variable separately. The *P* values presented were under the null hypothesis that “the coefficients of all interaction terms equal zero”.

**
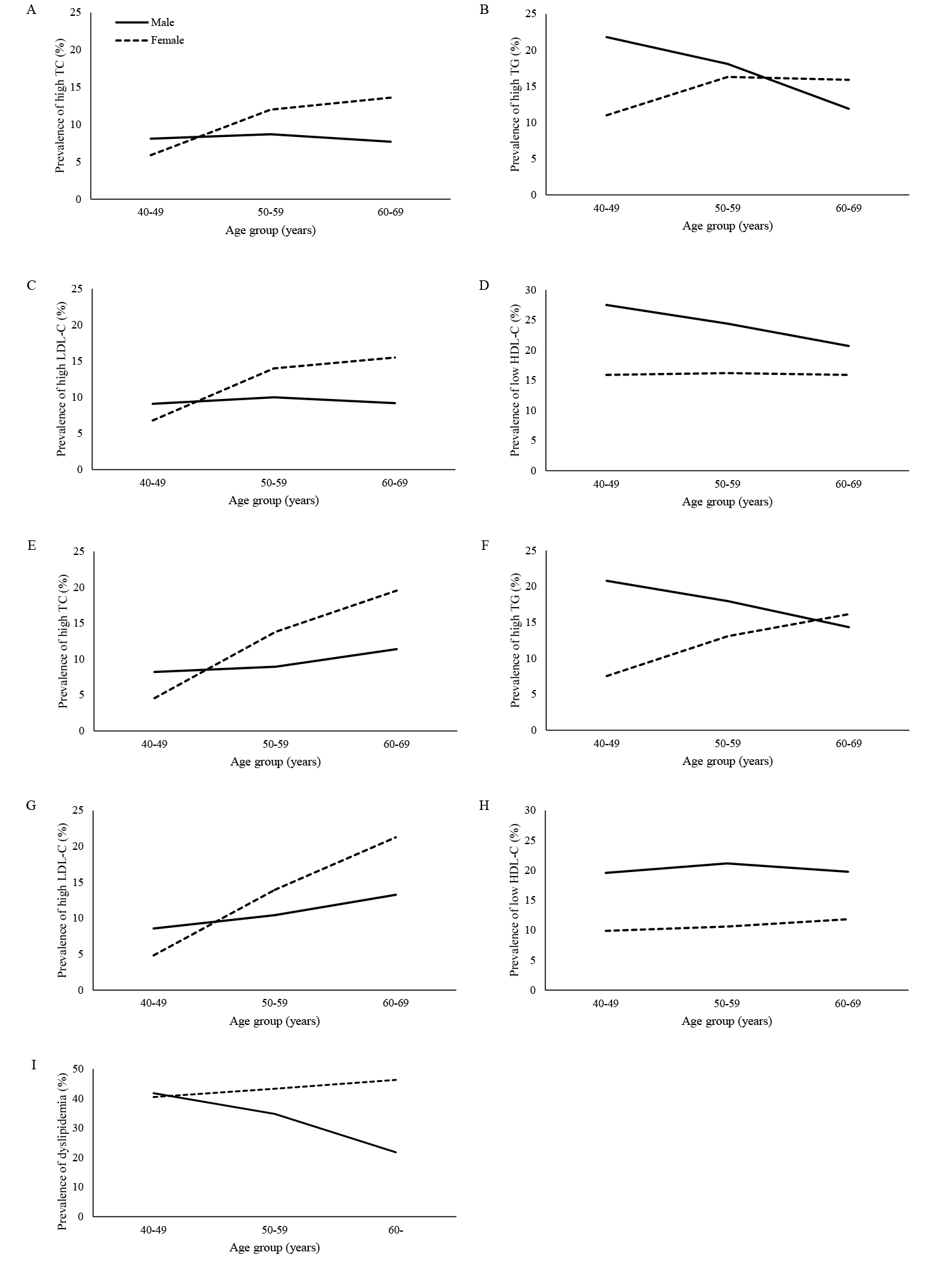
**

**Supplementary Figure 3. The prevalence of high TC, high TG, high LDL-C and low HDL-C in individuals 40-69 years in the 2013–2014 China Chronic Disease and Risk Factor Surveillance (CCDRFS), The China National Survey of Chronic Kidney Disease (CKD) and dyslipidemia investigation in Chongqing ^a^.**

A: Age and gender distribution of high TC in participants aged 40-69 years in the CCDRFS study^1^.

B: Age and gender distribution of high TG in participants aged 40-69 years in the CCDRFS study^1^.

C: Age and gender distribution of high LDL-C in participants aged 40-69 years in the CCDRFS study^1^.

D: Age and gender distribution of low HDL-C in participants aged 40-69 years in the CCDRFS study^1^.

E: Age and gender distribution of high TC in participants aged 40-69 years in the CKD study^2^.

F: Age and gender distribution of high TG in participants aged 40-69 years in the CKD study^2^.

G: Age and gender distribution of high LDL-C in participants aged 40-69 years in the CKD study^2^.

H: Age and gender distribution of low HDL-C in participants aged 40-69 years in the CKD study^2^.

I: Age and gender distribution of dyslipidemia in participants aged over 40 years in the dyslipidemia investigation in Chongqing^3^.

^a^ Prevalence data was extracted from the original articles of the CCDRFS study, the CKD study and dyslipidemia investigation in Chongqing.

**REFERENCE**

1. Zhang M, Deng Q, Wang L, et al. Prevalence of dyslipidemia and achievement of low-density lipoprotein cholesterol targets in Chinese adults: A nationally representative survey of 163,641 adults. International Journal of Cardiology. 2018;260:196-203.

2. Pan L, Yang Z, Wu Y, et al. The prevalence, awareness, treatment and control of dyslipidemia among adults in China. Atherosclerosis. 2016;248:2-9.

3. Qi L, Ding X, Tang W, Li Q, Mao D, Wang Y. Prevalence and Risk Factors Associated with Dyslipidemia in Chongqing, China. Int J Environ Res Public Health. 2015;12:13455-65.
